# Molecular characterization of human respiratory syncytial virus in Mexico (season 2023–2024) through whole-genome sequencing

**DOI:** 10.1038/s41598-025-13061-9

**Published:** 2025-07-28

**Authors:** Evelyn Rivera-Toledo, Fidencio Mejıa-Nepomuceno, Enrique Mendoza-Ramırez, America Vera-Jimenez, Eduardo Becerril-Vargas, Victor Hugo Ahumada-Topete, Manuel Castillejos-Lopez, Francisco Bernardo Perez-Orozco, Geovanni Benitez, Miguel Ángel Salazar-Lezama, Josue Daniel Cadeza-Aguilar, Emma Garcia-Colin, Claudia Garrido-Galindo, Justino Regalado-Pineda, John P. Collins, Xiang-Jun Lu, J. Kenneth Wickiser, Joel Armando Vazquez-Perez

**Affiliations:** 1 Departamento de Microbiología y Parasitología, Facultad de Medicina, Universidad Nacional Autónoma de México, Ciudad Universitaria,Coyoacán, Mexico City, 04510 Mexico; 2 Instituto Nacional de Enfermedades Respiratorias “Ismael Cosío Villegas”, Mexico City, Mexico; 3 Global Alliance for Preventing Pandemics, Mailman School of Public Health, Columbia University, New York, NY USA; 4 Department of Population and Family Health, Mailman School of Public Health, Columbia University, New York, NY USA

**Keywords:** Human respiratory syncytial virus, Whole-genome sequencing, Phylogeny, Lineages., Virology, Viral infection

## Abstract

Human respiratory syncytial virus (hRSV) is a one of major cause of severe acute respiratory infection (SARI) in young children and the elderly. Because genomic surveillance of hRSV is essential to understand viral evolution and the genetic variation that may affect transmissibility and pathogenicity, we sequenced complete genomes of hRSV-A and B from season 2023–2024, isolated from pediatric and adult patients with SARI. One hundred pediatrics and 43 hospitalized adult and 14 non-hospitalized adult patients testing positive for hRSV were enrolled. Libraries of whole hRSV genomes were generated and sequenced on a MiSeq platform. Phylogenetic analysis and maximum likelihood trees were constructed with the 64 hRSV-A and 29 hRSV-B sequences obtained in our study. Additionally, we analyzed the list of non-synonymous substitutions and their frequencies for each of the eleven viral proteins. hRSV-A was prevalent (68%) and principally affected children under five years old. The hRSV-A isolates belonged to the A.D lineage with sub-lineages A.D.1.5, A.D.1.8, A.D.3, and A.D.5.2 observed. The hRSV-B subgroup was less diverse since the dominant sub-lineage was B.D.E.1. Amino acid substitutions per viral isolate for each of the eleven viral proteins indicated higher variability in hRSV-A compared to hRSV-B. As expected, we observed a high diversity of substitutions in proteins G, F and L. Multiple lineages and a high mutation rate were identified in hRSV-A during winter season 2023–2024 in Mexico. The increasing availability of whole hRSV genome sequences will enhance the surveillance of specific genetic substitutions, contributing to a better understanding of viral evolution and the effectiveness of prophylactic and diagnostic strategies.

**Supplementary Information:**

The online version contains supplementary material available at 10.1038/s41598-025-13061-9.

## Introduction

Human respiratory syncytial virus (hRSV) is an enveloped virus from the *Pneumoviridae* family, which is a leading etiology of acute lower respiratory tract infection (ALRTI) in children under five years of age. hRSV infection resulted in approximately 33.0 million ALRTI, 3.6 million hospitalizations and 26,300 in-hospital deaths in 2019^1^. Annual hRSV-related hospitalizations are estimated between 356,000 and 466,000 and up to 33,000 in-hospital deaths in adults over 65 years of age in industrialized countries^2^.

The hRSV genome consists of ten genes encoded in a single-stranded RNA of approximately 15,200 nucleotides (nt) in length that expresses eleven proteins: NS1, NS2, N, P, M, SH, G, F, M2-1, M2-2 and L. The G and F envelope glycoproteins mediate viral attachment and entry to host cells, respectively, and are major targets of the immune response. The G protein is expressed as a precursor of 32 kDa that is modified by N- and O-glycosylation becoming a mature protein of 80–90 kDa^3^. The heavily glycosylated sequence is arranged as an ectodomain comprising two highly variable mucin-like domains connected by a central conserved region (aa 163–189) that includes a highly conserved sequence (aa 164–176), and four cysteines maintained in all viral strains^4,5^. The F glycoprotein is synthesized as a precursor, F(0), which is cleaved by a furin-like protease into two subunits, F1 and F2. F1 contains the fusion peptide that mediates virus entry through fusion step, following the interaction with the viral receptor^6^. Therefore, F exists in a metastable prefusion conformation and a highly stable postfusion structure. Six antigenic sites have been identified in F (denoted as ø–V), of which sites ø and V are exclusively found in the prefusion state^3^. Human neutralizing antibodies with high potency target the prefusion antigenic sites. As a result, the recently approved prophylactic monoclonal antibody Nirsevimab, and vaccines Abrysvo and Arexvy were designed to target the prefusion conformation of F^7^.

Two antigenic subgroups of hRSV A and B are recognized based on cross-neutralization assays and the sequence variation of the G gene, which exhibits the highest variability among the viral genes^8^. Since its first isolation in 1956, this virus has undergone genetic diversification, leading to the identification of emergent genotypes and lineages. A 60-nt duplication (20 amino acid) in the G gene of hRSV B was identified in the period of June to August 1999 in Buenos Aires, Argentina^9^. Also, in season 2010–2011 in Ontario, Canada, a duplication of 72-nt (24 amino acid) in the G gene of an hRSV-A isolate was observed^10^. Currently, both variants are circulating globally dominant and continue to evolve. Standardized criteria for the classification and genotyping of hRSV are essential in molecular epidemiology for monitoring the global circulation of variants. Recently, Goya et al., proposed a phylogenetic classification using whole-hRSV genome sequences and a system based on amino acid markers to define distinct lineages^11^. Although classification can be accomplished using the entire G and F genes sequences only, comparative studies, emerging lineages detection and phylogenetic analysis require whole genome data generated through WGS.

In Mexico, hRSV is the leading cause of hospitalization in children under two years of age during the winter season, and also contributes significantly to morbidity and admission rates among older adults. Although new therapies like palivizumab have been available in Mexico for more than a decade, their use remains inconsistent across at risk groups. More recently, two hRSV vaccines—one for adults and one for pregnant women—have been approved for use but are not yet accessible in the country. Similarly, nirsevimab a monoclonal antibody for infant prophylaxis against hRSV, has not yet been registered in Mexico^12^.

Because the general population in Mexico has minimal access to monoclonal antibodies and no exposure to hRSV vaccines, continuous whole genome surveillance of hRSV is essential to track genetic variants that may affect transmissibility or pathogenicity and to evaluate the potential emergence of immune escape variants. Such surveillance is critical both during the current pre-vaccine period and after vaccine introduction. To date, no studies have reported the molecular epidemiology of hRSV in Mexico using whole genome sequences. Here, we report the whole genome sequences of hRSV subtypes A and B collected during the 2023–2024 season from pediatric and adult patients presenting with severe acute respiratory infection.

## Methods

### Sample selection

As part of a surveillance program at the Instituto Nacional de Enfermedades Respiratorias (INER), nasopharyngeal swabs of individuals presenting cases of Influenza-Like Illness (ILI) and severe acute respiratory infection (SARI) are routinely collected. Patients with acute respiratory disease or chronic lung disease exacerbation requiring hospitalization, as well as ambulatory cases were sampled to be tested. One hundred and forty-three hospitalized patients, comprising 100 were children and 43 adults, as well as 14 non-hospitalized adult patients were enrolled in this study. All patients were confirmed by the RT-qPCR diagnostic kit Respiratory Panel Filmarray (BioMérieux, France). hRSV subgroup identification (subtyping) and cycle threshold (Ct) values were determined by RT-qPCR, using in-house designed primers for detection of the nucleocapsid (N) gene^13^.

### Viral RNA extraction and whole genome amplification

Viral RNA was extracted from 200 µl of nasal swabs in viral transport media (VTM), using the QIAamp Viral RNA mini kit (QIAGEN). The whole genome was amplified simultaneously and directly from clinical samples, using panel of primers modified and optimized for multiplex PCR^14^. Follow the indications of the protocol, whole genome amplification was performed in samples with a high viral load (Ct<25) to facilitate sequencing. The primers were used to cover the complete hRSV genome (both A and B subtypes) by splitting into two pools of non-consecutive amplicons. An alternative method was used for hRSV-A positive samples from winter season 2022–2023, alternative methodology was used^13^. For the hRSV-B samples COI-5 and COI-7, which were identified as co-infections by initial PCR testing, the VirCapSeq-VERT (VCS) protocol was utilized to sequence the whole genomes of all vertebrate viruses contained in the samples^15^. VCS data were analyzed with the Rapid Identification of Microbes (RIM), a custom viral metagenomics pipeline (http://rimv1.gapp-cii.org/)^16^.

### Libraries Preparation and sequencing

Libraries of hRSV complete genome were generated using the reagents of the Covid-Seq kit (Illumina, San Diego, CA, USA). Libraries were sequenced on a MiSeq sequencing platform using a 2 × 150-cycle to obtain paired-end reads (Illumina, San Diego, CA, USA). The DRAGEN COVIDSeq Targeted Microbial Pipeline on BaseSpace Sequence Hub performed the analysis, mapping, and consensus (coverage depth threshold = 10X). For downstream analyses, samples with more than 80% of coverage and less than 500 ambiguous bases (Ns) were used (Table S1). For COI-5 and COI-7, libraries were prepared with Twist library preparation and fast hybridization reagents (Twist Bioscience, San Francisco, CA, USA), and sequenced on a NextSeq 2000 instrument using a 2 × 150-cycle to obtain single-end reads (Illumina, San Diego, CA, USA). Mapping and consensus were carried out with the RIM pipeline.

### Phylogenetic and mutations analysis

To perform phylogenetic analysis, we analyzed 1,029 whole hRSV-A genomes. This included 874 sequences from various U.S. states from 2022 to 2024, 25 sequences from Panama from 2022 to 2023 and 49 sequences from Canada, all obtained from the GISAID database. Additionally, we analyzed the 81 hRSV-A whole genome sequences from this study, including 19 sequences from winter season 2022–2023. For hRSV-B, we included 495 whole genomes: 453 sequences from different U.S.states from 2021 to 2024 and 11 from Argentina, all available on the GISAID. We also included the 31 whole hRSV-B genome sequences from this study, including 2 sequences from 2022 to 2023 winter season. A maximum likelihood tree was constructed for the whole genome sequence using MEGA 10.0. The General Time-Reversible model was selected with five-parameter gamma-distributed rates and 1000 bootstrap replicates. Tree annotations were generated using FigTree^17^. hRSV-A and hRSV-B clades were assigned using the real-time phylogenetic analysis in Nextclade^18^. Non-synonymous substitutions were identified using reference sequences for hRSV-A (A/England/397/2017 accession number EPI_ISL_412866) and hRSV-B (B/Australia/VIC-RCH056/2019 accession number EPI_ISL_1653999) in Nextclade. The nomenclature established in 2023 uses a combination of letters and numbers to designate lineages in a hierarchical fashion^19^. Samples with unusually long branches were eliminated from the phylogenetic analysis.

### Statistical analysis

Statistical analyses were performed with GraphPad Prism version 10. The Shapiro-Wilk test was used to assess data normality, with a p-value < 0.05 indicating deviation from a normal distribution. Non-parametric data were analyzed with the Kruskal-Wallis test to determine differences between groups. A p-value < 0.05 was considered statistically significant.

## Results

During the 2023–2024 season, a total of 100 pediatric patients (aged 1 month to 17 years) and 43 adult patients (mean age 55 ± 18 years) with confirmed hRSV infection by RT-qPCR were hospitalized at the INER.

Comorbidities were identified in 68% of children, with the most common being malnutrition (18%), followed by recurrent wheezing (14%), gastroesophageal reflux diseases (12%), asthma (11%) and allergic rhinitis (11%) (Table 1).

**Table 1:** Demographics of the pediatric patients.

Pediatric hospitalized patients	*N* = 100	(100%)
Boys	43	43%
Girl	57	57%
Age (years)		
0–2	47	47%
> 2–6	40	40%
> 6–17	13	13%
Comorbilities	68	68%
Malnutrition	18	18%
Recurrent wheezing	14	14%
Gastroesophageal Reflux Disease	12	12%
Asthma	11	11%
Allergic Rhinitis	11	11%
Recurrent pneumonia	10	10%
Bronchopulmonary Dysplasia	5	5%
Shock	4	4%
Asphyxia	3	3%
AOS	3	3%
Atopic dermatitis	3	3%
Tracheobronchomalacia	2	2%
Epilepsy	2	2%
Other relevant antecedents upon admission		
Breastfeeding	76	76%
Tobacco smoke exposure	26	26%
Vaccination compliance	23	23%
Prematurity Nutritional status	19	19%
Eutrophic	77	77%
Malnutrition	18	18%
Overweight	5	5%
Days start symptoms to addition	4 (3.0–6.0) Q1-Q3	
Hospitalization days	7 (5.0–10) Q1-Q3	
Viral respiratory panel results		
hRSV-A	68	68%
hRSV-B	20	20%
Unsubtypeable	12	12%
Coinfections	48	48%
Rhinovirus/enterovirus	24	24%
Metaneumovirus	12	12%
Adenovirus	7	7%
Influenza AH1N1	3	3%
Other coronavirus	2	2%
Parainfluenza type 1	2	2%
Parainfluenza type 2	2	2%
Parainfluenza type 3	2	2%
Parainfluenza type 4	2	2%
SARS-CoV-2	2	2%
Influenza A H3N2	1	1%

Subgroup identification was performed in 88/100 pediatric samples, of which 68% were positive for hRSV-A and 20% positive for hRSV-B. Additionally, 12% of pediatric samples tested positive for hRSV by Viral Respiratory Panel (Filmarray) but could not be further subtyped by the specific RT-qPCR assay.

In hospitalized adults, the most common comorbidities were chronic lung disease, asthma, COPD, IFD, obesity and type 2 diabetes.

### hRSV phylogeny

Whole hRSV genome sequencing was achieved for 58 respiratory samples from pediatric patients and 35 from adults (Table 2, Supplementary materials). Of these, 64 correspond to hRSV-A and 29 to hRSV-B. The monthly distribution of these sequences in the winter season 2023–2024 were as follows: October 2023: 30, November 2023:42, December 2023:15, January 2024:5 and February 2024:1. Due to the absence of prior genotyping report based on whole genome in Mexico, we used 19 sequences obtained during the 2022–2023 winter season to support the phylogenetic analysis and lineages classification. (GISAID accession number EPI_ISL_19500979- EPI_ISL_19501009 and EPI_ISL_19504707-EPI_ISL_19504788).

**Table 2:** Total hRSV-A and hRSV-B isolates sequenced from pediatric and adult samples 2023–2024 winter season.

hRSV subgroup	Pediatric (No.)	Adult (No.)	Total
A	41	23	64
B	17	12	29
Total	58	35	93

**Fig. 1:**
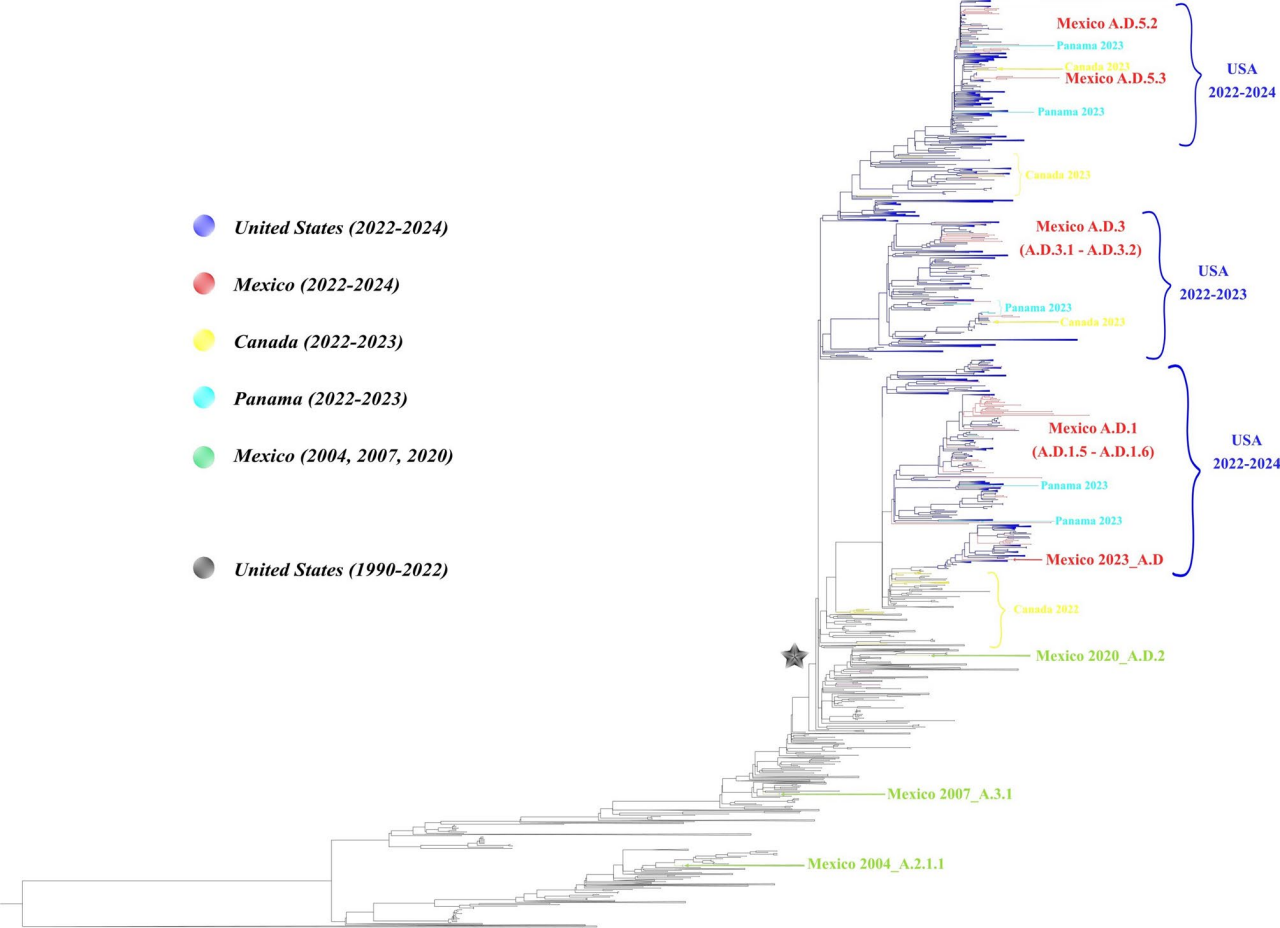
Maximum likelihood (ML) phylogenetic tree for RSV-A complete genome. ML tree from 1029 hRSV-A viruses registered in GISAID including 81 Mexican sequences from 2022–2024 of this study (colored in red). Branches indicate the lineage classification and the black star indicates A.D lineage. Scale bar indicates substitutions per site.

**Fig. 2:**
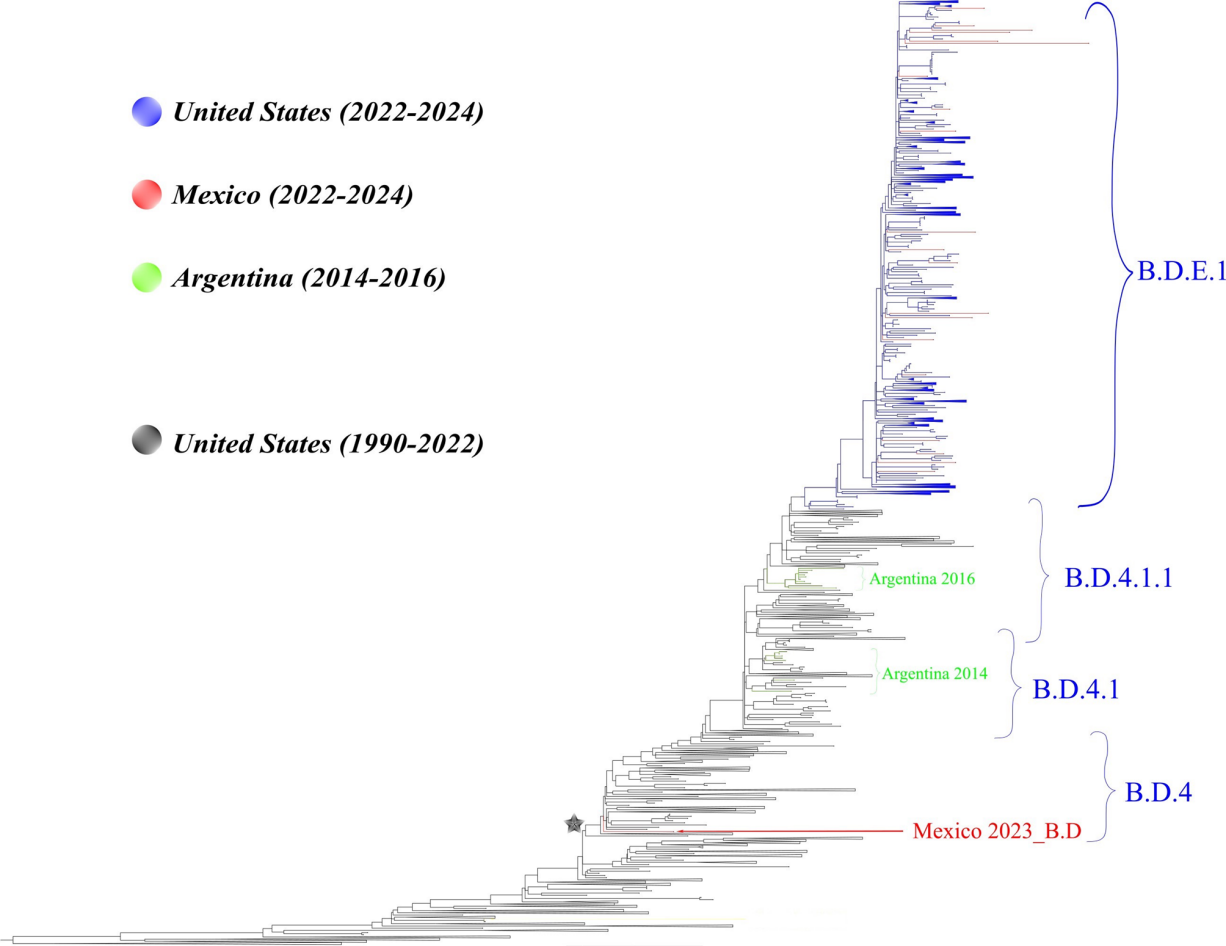
Maximum likelihood (ML) phylogenetic tree for RSV-B complete genome. ML tree from 495 hRSV-B viruses registered in GISAID including 31 Mexican sequences from 2022–2024 of this study (colored in red). Branches indicate the lineage classification and the black star indicates B.D lineage. Scale bar indicates substitutions per site.

Phylogenetic analyses indicated that all hRSV-A isolates from 2022 to 2024 belonged to the A.D lineage (which carry the duplication of 72 nt in the G gene) and ten sublineages (Fig. 1). The sublineages A.D.1.5, A.D.1.8, A.D.3, and A.D.5.2 were observed (66/81 isolates, Table 3), mainly in children under 5 years old. The analysis showed that Mexican sequences formed 3 different groups: A.D.1 and its sub-lineages; A.D.3 and its sub-lineages, and A.D.5 and its sub-linages (Fig. 1). Remarkably, two sequences of 2023 belong to clade A.D. which is the ancestor of the newer sub-lineages. Each group clustered with U.S. sequences from 2022 to 2024, 2023–2024 and 2022–2024 respectively (Fig. 1). The hRSV-B sequences were less diverse (Fig. 2), with all belonging to the B.D lineage (characterized by the 60 nt duplication in the G gene) and the dominant sub-lineage was B.D.E.1 (28/29 isolates). Mexican sequences (B.D.E.1 lineage) clustered with U.S. sequences from 2022 to 2024 (Fig. 2). Lineage-defining amino acids in Mexican sequences were present mainly in G, L and F genes and are summarized in Table 4.

**Table 3:** Frequency of positive samples for the different hRSV-A lineages by age group, 2022–2023 and 2023–2024 winter season.

Lineage	All, *n* = 81 No.(%)	0–5 years No.(%)	6–17 years No.(%)	22–54 years No.(%)	58–81 years No.(%)
A.D.	2 (2.5)	1(50.0)	0 (0.0)	1(50.0)	0 (0.0)
A.D.1	4 (5.0)	3(75.0)	1(25.0)	0 (0.0)	0 (0.0)
A.D.1.4	3 (3.7)	2(66.6)	0 (0.0)	1(33.3)	0 (0.0)
A.D.1.5	27(33.3)	13(48.1)	4(14.8)	3(11.1)	7(26.0)
A.D.1.7	1(1.2)	1(100.0)	0 (0.0)	0 (0.0)	0 (0.0)
A.D.1.8	10(12.3)	7(70.0)	1(10.0)	1(10.0)	1(10.0)
A.D.3	10 (12.3)	4(40.0)	2(20.0)	3 (30.0)	1(10.0)
A.D.3.2	1(1.2)	0(0.0)	0(0.0)	0 (0.0)	1(100.0)
A.D.3.3	2(2.5)	1(50.0)	0(0.0)	1(50.0)	0 (0.0)
A.D.5.2	19 (23.5)	9(47.4)	3(15.8)	4 (21.0)	3(15.8)
A.D.5.3	2(2.5)	1(50.0)	0(0.0)	1 (50.0)	0(0.0)

**Table 4:** Mutations defining A.D and B.D hRSV-A clades 2022–2023 and 2023–2024 winter season.

Lineages	Mutations
hRSV-A	
A.D.1	G: L142S, T320A, L:N413D, T179S, I1653V, K1661N, F1725G
A.D.1.4	G: A122V, L:I1656T, G1735D, V:1934I
A.D.1.5	G: T319I, F:V127I, M2-1:N74S, T180A, M2-2:T1M
A.D.1.7	F:157 V, M2-2:I78V
A.D.1.8	G: Y280H, L:N2025S
A.D.3	G: T113I, V131D, N178G, H258Q, H266L, F:T12I, M2-2:T79A
A.D.3.2	NS1:I71V, P:S60P, M92I, G:F101S, I243T, L274P, Y304H, L:I1653V
A.D.3.3	P: G74E, G:R151Q, G178N, E263K, L:H2101Y
A.D.5	G: V303A, L:S1723G
A.D.5.2	M2-2: C26Y, L:E1725G
A.D.5.3	G: L314P, M2-2:D64E
hRSV-B	
B.D.E.1	G: S275P, Y285H, F:S389P, M2-2:D35N

### Amino acid substitutions in hRSV proteins

**Fig. 3:**
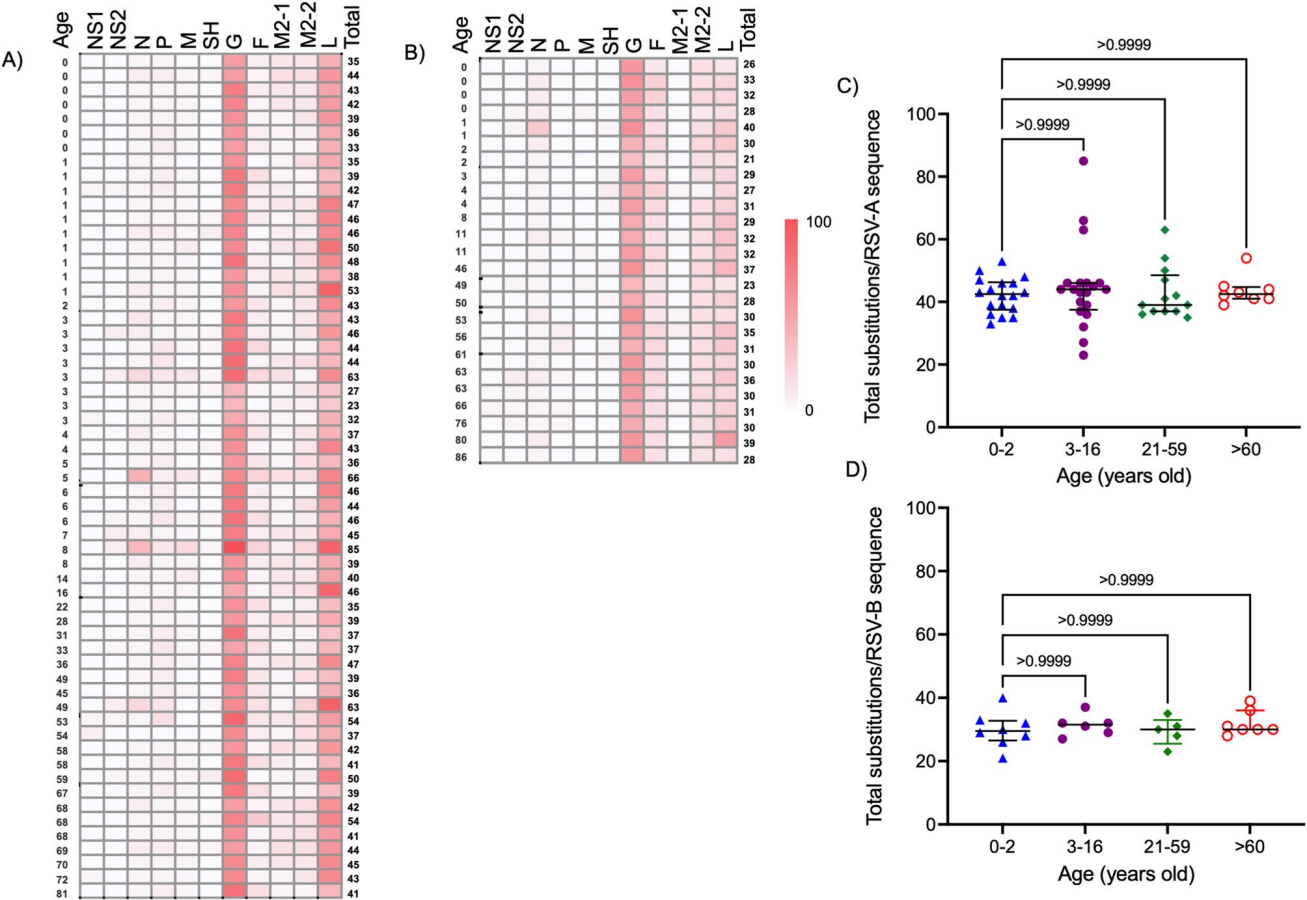
Total substitutions in hRSV proteins and association with age groups. The heat maps represent substitutions identified in (A) hRSV-A, and (B) hRSV-B. Each row corresponds to individual hRSV-A and hRSV-B isolates (59 and 26, respectively), whereas columns represent substitutions in each of the eleven viral proteins. The numeric value in the last column indicates total mutations per virus isolate. Age is indicated in the left side of the heat maps. (C) and (D) Total substitutions in hRSV-A and hRSV-B isolates, respectively, with respect to age group. Median with interquartile range is shown. Difference between groups was analyzed by Kruskal-Wallis test; *p* < 0.05 was considered statistically significant.

To avoid bias in the mutational analysis, 5 hRSV-A and 3 hRSV-B sequences with high numbers of substitutions in the L gene were eliminated. The total number of amino acid substitutions per hRSV isolate is presented in the heat maps in Fig. 3. Among the 59 hRSV-A sequenced samples from 2023 to 2024, a median value of 43 amino acid substitutions per viral genome (range 23–85, Fig. 3A) was found, while for the 26 hRSV-B positive samples the median value was 30 substitutions per viral genome (range 21–41, Fig. 3B). It was evaluated whether the number of substitutions were similar between hRSV sequences from the following different age groups: infants (0–2 years old), children and adolescents (3–16 years old), adults (21–59) and older adults (over 60 years). Results showed a tendency to increased number of substitutions in hRSV-A isolates from individuals in groups 0–5 and 3–16 years old, compared to the 21–59 years old group, although it was not statistically significant (Fig. 3C). The total number of substitutions between hRSV-B isolates was not significantly different between either age group (Fig. 3D). The highest variability was observed in the G glycoprotein (Fig. 3A and B) from both hRSV subgroups. Table 5 summarizes the total number of all the diverse substitutions identified in the eleven encoded proteins from virus sequences. Although the highest number of substitutions was identified in the L protein, the substitution density, which is related to the length of each protein, was higher in the protein G, followed by protein M2-2 in hRSV-A, and SH in hRSV-B. Interestingly, hRSV-A showed more variation than hRSV-B, as the calculated hRSV-A/hRSV-B substitution ratio was > 1 for each protein.

**Table 5:** Number and density of hRSV protein substitutions 2023–2024 winter season.

Protein	hRSV-A			hRSV-B			RSVA/ RSVB**
	Length (aa)	Substitution (total)	Substitution density*(%)	Length (aa)	Substitution (total)	Substitution density*(%)	
NS1	140	4	2.8	140	1	0.7	4.0
NS2	125	14	11.2	125	7	5.6	2.0
N	392	35	8.9	392	13	3.3	2.7
P	242	15	6.2	242	8	3.3	1.9
M	257	12	4.7	257	2	0.7	6.0
SH	65	9	13.8	66	6	9.1	1.5
G	322	110	34.2	311	56	18.0	1.9
F	575	39	6.8	575	24	4.2	1.6
M2-1	195	19	9.7	196	6	3.1	3.1
M2-2	91	23	25.3	91	8	8.8	2.9
L	2166	190	8.8	2167	63	2.9	3.0

* (No. of substitutions/protein length) $\:\times\:$100; **Ratio between the total number of substitutions per protein in hRSV-A and hRSV-B.

**Fig. 4:**
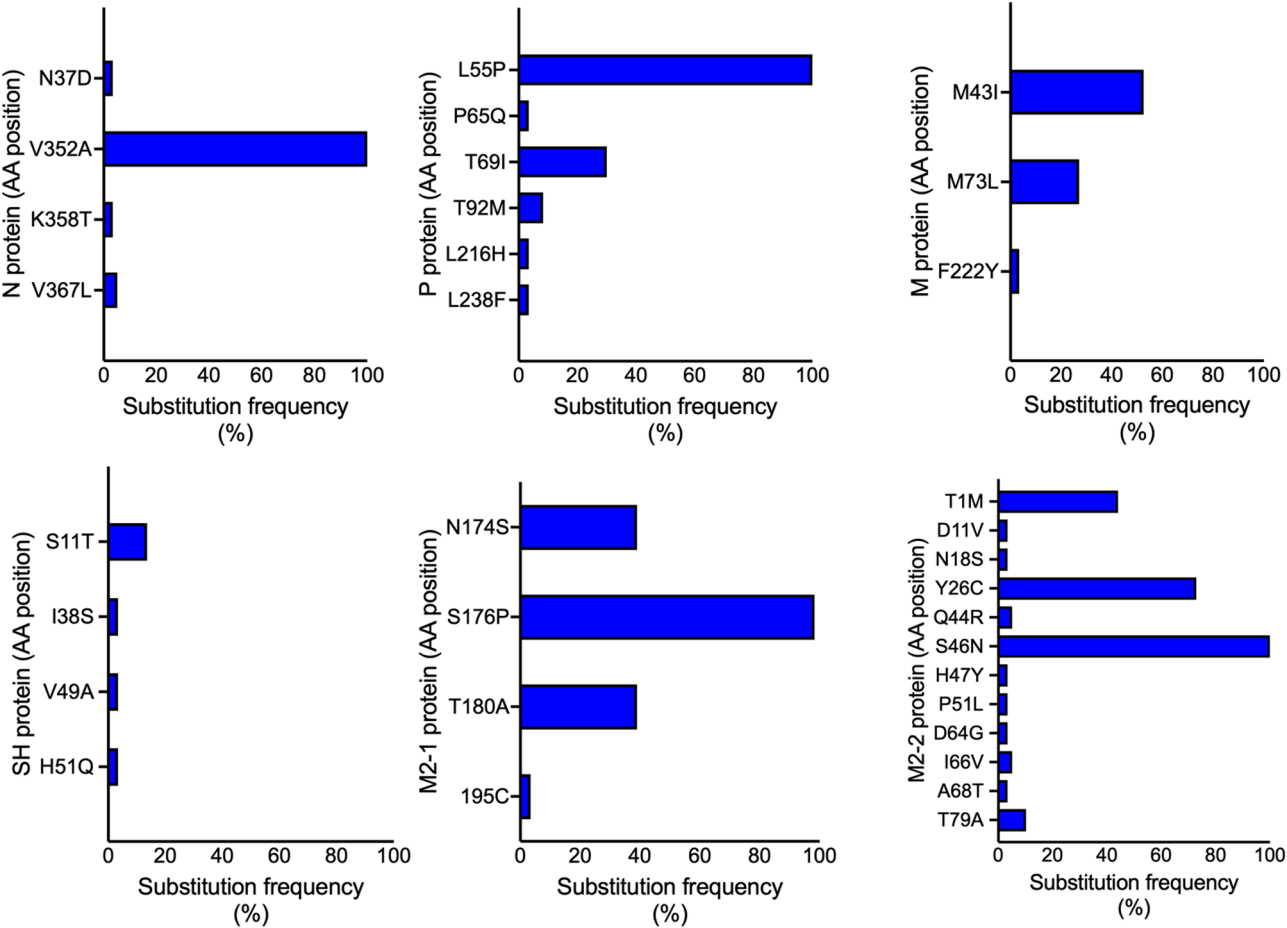
Substitutions in hRSV-A proteins. The bars represent amino acid positions with substitutions occurring at a frequency ≥3.4% (at least 2/59 sequences).

**Fig. 5:**
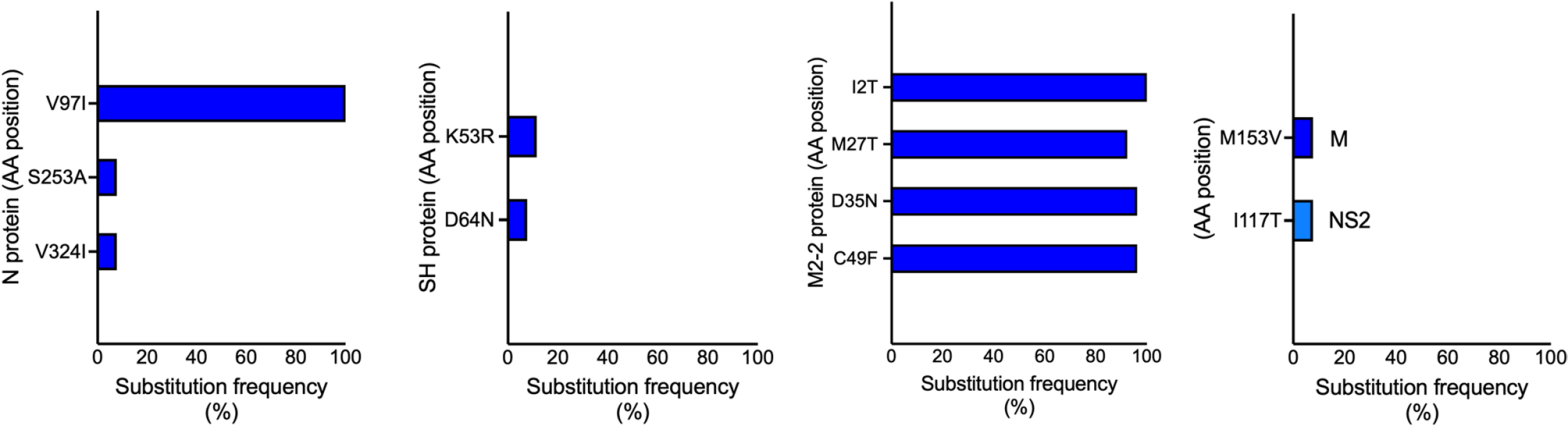
Substitutions in hRSV-B proteins. The bars represent amino acid positions with substitutions occurring at frequencies ≥7.7% (at least 2/26 sequences). Proteins M and NS2 each exhibited a single mutation with frequency ≥7.7%; both are represented in the same graph.

Several amino acid substitutions were unique to individual viral genome and all substitutions are reported (Additional_file_1). Figures 4 and 5 show amino acid substitutions identified in at least two viral genomes, particularly in proteins NS2, N, P, M, SH, M2-1 and M2-2. The lowest number and frequency of substitutions was observed in the NS1 and NS2 proteins for hRSV-A and for hRSV-B, the least variation was seen in NS1, M, P and M2-1 proteins (Additional_file_1). In contrast, substitutions with frequencies > 40% were detected in N, P, M, M2-1, and M2-2 proteins of hRSV-A, as well as in N and M2-2 proteins of hRSV-B (Figs. 4 and 5).

**Fig. 6:**
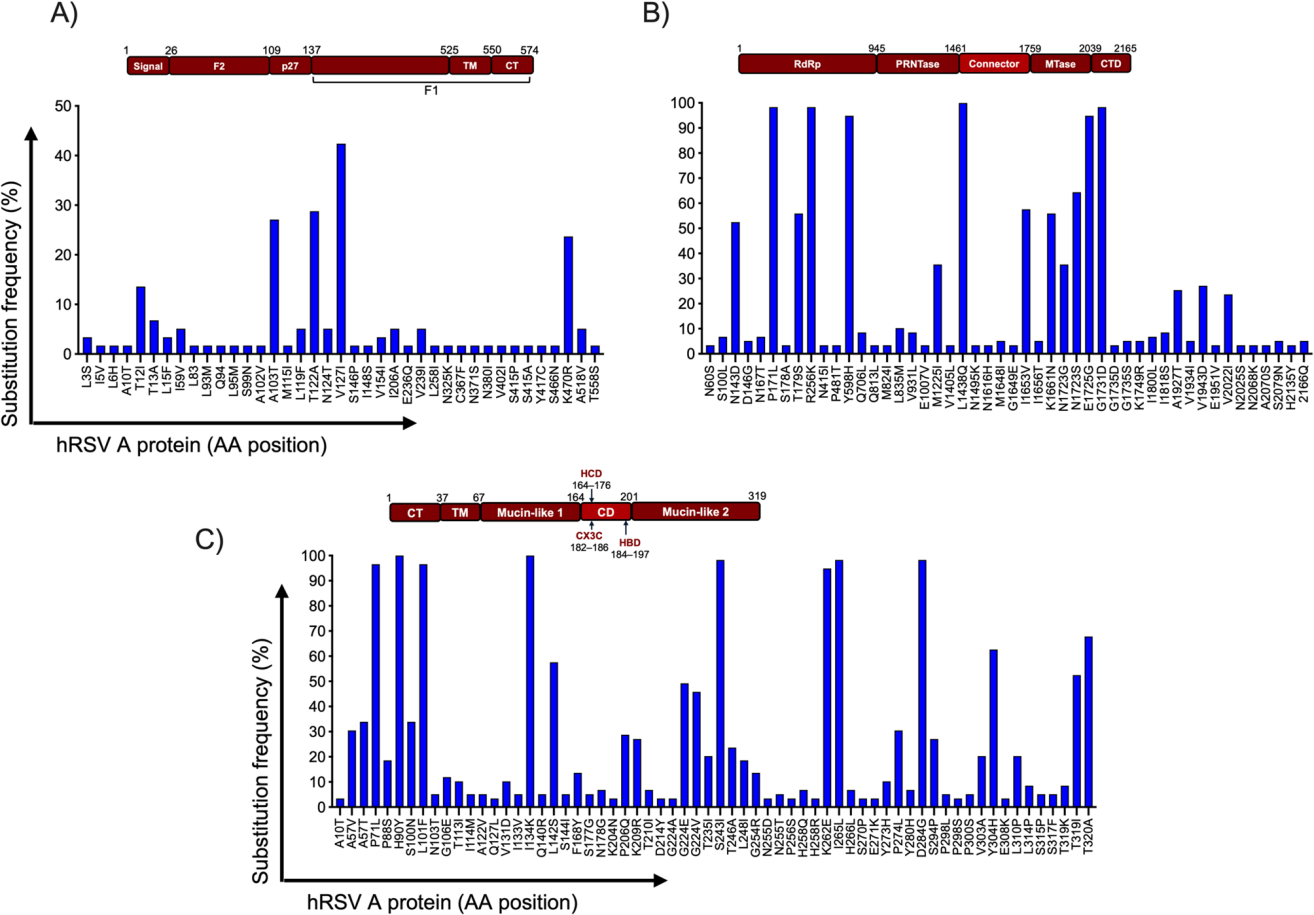
Substitutions in the hRSV-A envelope proteins and viral polymerase. The bars represent amino acid positions with specific substitution frequencies. (A) F protein, (B) viral polymerase (protein L), and (C) G protein. Graph bar (A) represents all mutations identified in the F protein of hRSV-A, whereas (B) and (C) show only mutations with frequency values ≥3.4%. The distribution of the main domains in each viral protein is also represented. Abbreviations: CT, cytoplasmic domain; TM, transmembrane domain; CD, conserved domain; HCD, highly conserved domain; HBD, heparin-binding domain; RdRp, RNA-dependent RNA polymerase; PRNTase, polyribonucleotidyl transferase or capping domain; MTase, methyltransferase domain; CTD, C-terminal domain.

**Fig. 7:**
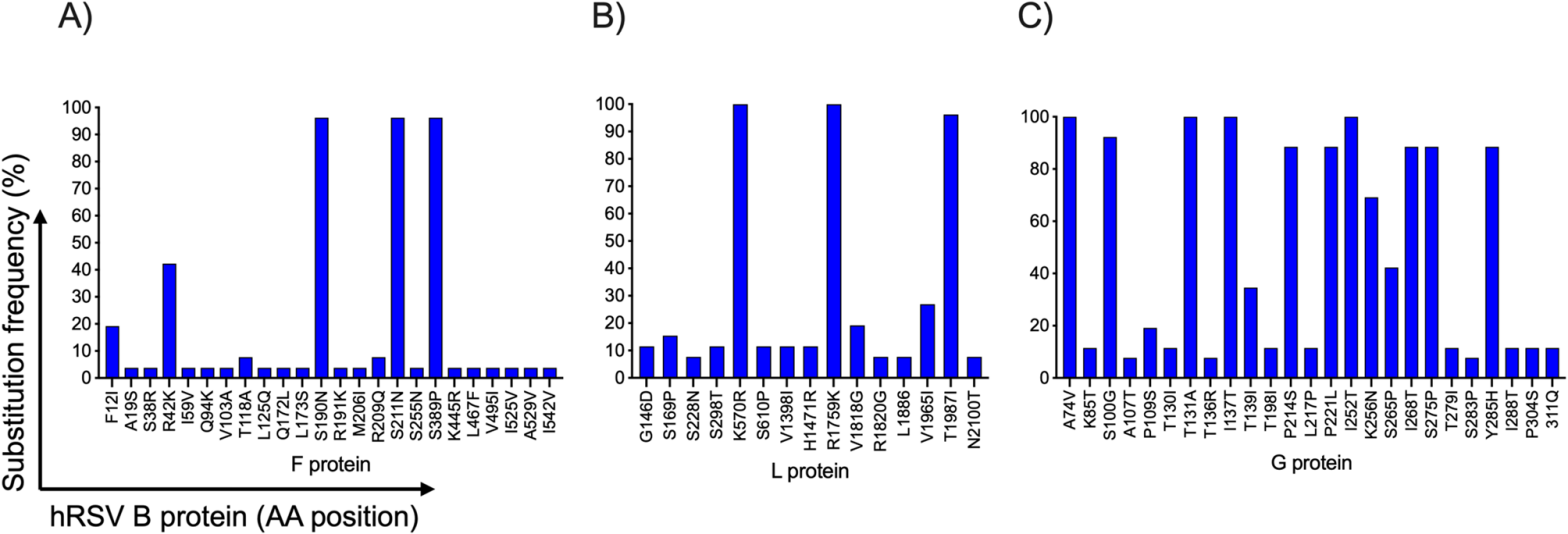
Substitutions in the hRSV-B envelope proteins and viral polymerase. The bars represent amino acid positions with specific substitution frequencies. (A) F protein, (B) viral polymerase (protein L) and (C) G protein. Graph bar (A) represents all mutations identified in the F protein of hRSV-B, whereas (B) and (C) display only mutations with frequencies ≥7.7%.

Substitutions in the envelope glycoproteins that mediate viral entry and in the polymerase were diverse, with many occurring at frequencies higher than 50% (Fig. 6), except in the F protein of hRSV-A. More frequent amino acid substitutions in the F glycoprotein of hRSV-A were localized in the signal peptide region (T12I, T13A, L15F), F2 (I59V, A103T), p27 (L119F, T122A, N124I, V127I), and in the ectodomain (K470R, A518V) of F1 (Fig. 6A). Low frequency substitutions were identified in the fusion peptide (S146P, I148S, V154I), the antigenic site ø (I206A) and the antigenic site II, specifically in the binding site of Palivizumab (L258I). The F protein of hRSV-B showed less variability than that of hRSV-A, although some substitutions were detected with frequencies close to 100%. Of those, three were localized to the ectodomain of F1 (S190N, S211N, S389P) and particularly, S211N was localized in the antigenic site ø (Fig. 7A). Two other low frequency substitutions in site ø were M206I and R209Q.

In the G glycoprotein, most substitutions were localized to the mucin-like regions 1 and 2. Interestingly, G from hRSV-A showed three substitutions in the central conserved region (F168Y, S177G, N178G) of which, F168Y was within the highly conserved domain (HCD, Fig. 6B). In hRSV-B the substitution T198I, also localized in the central conserved region was observed with frequency of 11.5% (Fig. 7B).

The viral polymerase displayed most substitutions in the RdRp domain of both hRSV-A and hRSV-B with 69 and 32 substitutions, respectively. In contrasts, the least variable region was the catalytic domain with MTase activity (Figs. 6C and 7C; Supplementary material). Positions R1339 and G1855 have been described as relevant for capping and MTase activity, respectively^20^. We identified substitutions R1339P and G1855R in one of the Mexican hRSV-A sequence (Additional_file_1).

Finally, there were no deletions or insertions found in hRSV-A and hRSV-B sequences.

## Discussion

Hospitalizations due to acute hRSV lower respiratory tract infections during the 2023–2024 season showed that infants under five years of age were the primary affected demographic group.

hRSV-A exhibited a higher prevalence compared to hRS-B in both pediatric and adult patients. Particularly, infections by hRSV-A outnumbered those by hRSV-B by 3.4-fold in pediatric patients and approximately 1.7-fold in adults (Tables 1 and 2). Previous studies have documented that hRSV-A replicates faster and at higher titers (up to 2 log_10_ PFU/ml) than hRSV-B in airway human epithelial cell cultures^21,22^. However, similar viral loads have been found in respiratory samples from infants infected with either subgroup A or B, as measured by RT-qPCR or viral titration^23,24^, suggesting that factors besides replication rate contribute to the higher prevalence of hRSV-A.

Phylogenetic analysis demostrated that the prevalent lineages of hRSV-A were A.D.1.5, A.D.1.8, A.D.3, and A.D.5.2, while for hRSV-B, the predominant lineage was B.D.E.1. Studies conducted in three different countries reported the prevalence of the following clades after season 2021–2022: A.D.3, A.D.5, A.D.5.2 and B.D.E.1 in USA (Minnesota); A.D.3, A.D.5.2, B.D.4.1.1 and B.D.E.1 in China (Beijing); and A.D.1, A.D.3.1, A.D.5.2 and B.D.E.1 in Italy (Sicily)^25–27^. Accordingly, A.D.3, A.D.5.2 and B.D.E.1 may have global distribution^27^. Notably, the study from Beijing, China reported that B.D.E.1 became prevalent in November 2023 and affected a significantly higher proportion of patients ≥ 60 years (3-fold increase) than did its parent lineage B.D.4.1. However, B.D.E.1 was mostly associated with development of upper respiratory tract infections and non-severe pneumonia, conversely to the severe diseases caused by the previously dominant lineages A.D, A.D.3 and B.D.4.1^27^.

Amino acid substitutions per viral genome and the substitution ratio (hRSV-A/hRSV-B) for each protein indicated higher variability in hRSV-A compared to hRSV-B. Total substitutions showed a tendency to increase in the hRSV-A isolates infecting the 0–16- year-old age group than in the 21–59-year-old group. This difference may be at least partially explained by the prevailing circulation of the hRSV-A in children, a host population with a naïve or minimally experienced immune system. This condition facilitates higher viral replication, which in turn can lead to an increased mutation rate^28,29^. The absence of RSV-B cases in the 12–45 age range (Fig. 3A and B) may reflect the limited number of total hRSV-B samples or potentially a lower susceptibility to hRSV-B infection in this age group, associated with asymptomatic or mild infections that did not require medical attention or sample collection.

As expected, we observed a high diversity of substitutions in G, F and L proteins. However, all substitutions in the F protein of hRSV-A showed frequencies < 40%, whereas many substitutions in G and L proteins displayed frequencies of up to100%.

The F protein contains six potential N-glycosylation sites located at positions N27, N70, N116, N120, N126 and N500. N116 and N126 are within the p27 fragment, which is cleavaged by furin-like cellular proteases^30^, while N120 is not conserved among different hRSV isolates^31^. Both, the double mutant N27Q/N70Q and the individual N500Q impair membrane fusion^31^. We did not identify substitutions in the N-glycosylation sites of our viral isolates. However, the substitution T122A (with a frequency of 28.8%), located in the consensus N-glycosylation sequence N-X-T/S was observed. It has been suggested that T122A might reduce the glycosylation at N120, although this likely has no significant impact on fusion activity^31^.

On the other hand, substitutions were detected in the F protein located within the antigenic sites ø and II. Particularly, the low frequency substitutions L258I was found in the Palivizumab binding site (residues 258 to 275) of hRSV-A. Zhu et al., reported 46 polymorphic sites in the extracellular region of the F protein of clinical isolates from children without prophylactic treatment^32^. Using microneutralization assays, it was determined that only the substitutions N262D and S275F conferred resistance to Palivizumab. These substitutions were identified in 2 of 145 hRSV A isolates by the authors and were considered as natural polymorphisms. In this study, changes in these positions were not detected.

Palivizumab resistance has been observed in 5–10% of immunocompromised infants with long-term infections and under treatment with Palivizumab. In such cases, substitutions N262D, K272E and S276N have been reported, although only the first two were associated with Palivizumab resistance^32,33^.

The recently approved prophylactic monoclonal antibody Nirsevimab targets a prefusion discontinuous neutralizing epitope within site Ø, spanning residues 62 to 69 and 196 to 212^34^. Although most residues within the binding site are conserved at a frequency of > 99%, a partial reduction in susceptibility to Nirsevimab has been associated with specific substitutions. These include N208D/S, K65Q/T and the dual substitution N67I/N208Y in hRSV-A, or dual substitutions K68N/N201S and K65Q/S211N in hRSV-B^35,36^.

In our study, we identified the substitution I206A (5.1%) in hRSV-A, and the substitutions M206I (3.8%), R209Q (7.7%), and S211N (96.2%) in hRSV-B. These substitutions within the Nirsevimab binding site have been documented as natural polymorphisms, and through microneutralization assays it has been determined that changes in the position 206 of the hRSV A-F protein do not modify the neutralization activity of Nirsevimab, while single mutations in positions 206 and 209 only partially reduce neutralization activity against hRSV-B isolates^36^. Given that the newly approved vaccines also target the prefusion conformation of the F protein, the variability we observed in antigenic sites, including low-frequency substitutions within sites Ø, raises the need to monitor for vaccine escape variants in Mexico where vaccines were approved in December 2024^37^.

Other substitutions in F protein, localized in the signal peptide and p27 showed frequencies > 13%. Consistent with the observations of this study, it has been previously reported that domains with the greatest number of non-synonymous changes and amino acid positions with higher entropy values are within the signal peptide, p27, heptad repeat domain 2, antigenic site ø, and the transmembrane domain^38^.

As predicted, the G glycoprotein showed high variability, primarily in the second mucin-like region. However, the substitutions F168Y, S177G and N178G in the central conserved domain (CCD) of hRSV-A and substitution T198I in hRSV-B were also identified. Attachment of hRSV to primary respiratory epithelial cells occurs by binding of the G protein to CX3CR1 and infection is attenuated in viruses lacking the G protein or with mutations in the CX3C motif^39^. Although the highly glycosylated domains of the G protein are poor immunogens, antibodies against epitopes within the CCD have been detected. These antibodies can induce antibody-dependent cellular cytotoxicity or block the CX3C–CX3CR1 interaction^40,41^. Furthermore, substitutions as 177Q and 177R in the CCD enhance G protein immunogenicity and induce IgG antibodies that inhibit the CX3C–CX3CR1 interaction, thereby reducing pulmonary cell infiltration and lung damage in mice^42^. In this study, the substitution S177G at a low frequency (3.7%) in hRSV-A isolates were identified. It would be of interest to evaluate if the serum from individuals infected with this variant can reduce hRSV infection of primary airway epithelial cells.

On the other hand, Li et al., previously reported that substitutions T113I, V131D, N178 G, H258Q and H266 L in the G protein are associated with decreased disease severity in hospitalized infants^43^. We found the same five substitutions in hRSV-A isolates, with frequencies ranging from 6.8 to 10.2%. Further studies are necessary to assess the impact of these substitutions on viral infectivity.

Regarding the L protein or RNA-dependent RNA polymerase, we identified 190 and 63 different non-synonymous substitutions in hRSV-A and hRSV-B isolates, respectively. This enzyme is multifunctional, as it not only participates in the transcription and replication of the viral genome but also exhibits polyribonucleotidyltransferase (PRNTase) activity to add the cap structure, as well as methylase activity to methylate the cap^44^. Certain mutations in the L protein have been identified in the context of studies with antiviral drugs that inhibit its enzymatic activity^45–47^. Substitutions associated with antiviral drug resistance were not identified in this study. However, substitutions R1339P and G1855R within the catalytic pocket of the capping domain and the SAM/SAH GxGxGx binding motif of the MTase domain, respectively were observed^20^. Both substitutions were present at a frequency of 1.7%.

Substitutions in hRSV proteins other than F, G and L have been less extensively studied. Nevertheless, the growing availability of complete hRSV genome sequences will facilitate the surveillance of specific substitutions and their frequencies, thereby contributing to a better understanding of viral evolution and the effectiveness of prevention and treatment strategies.

This study represents the first detailed genomic characterization of hRSV-A and hRSV-B isolates from hospitalized pediatric and adult patients in Mexico City during the 2023–2024 season, contributing to global surveillance efforts. These results confirm the previously described predominance of hRSV-A and its high variability in the G protein, but also show demonstrate the variability in the less-studied P, M2-1 and M2-2 proteins. The local circulation of globally distributed lineages such as A.D.5.2 and B.D.E.1 was observed, as well as the occurrence of substitutions within the antigenic sites Ø and II of the F protein. These mutations speak to the need for continuous monitoring to assess potential impacts on vaccine and monoclonal antibody effectiveness in our geographic region. Although age-stratified analysis did not reveal significant differences in substitution frequency, we observed a tendency toward a higher number of substitutions in viral genomes isolated from infants, which might reflect age-related differences in immune pressure. However, further studies with higher number of samples are necessary to validate this hypothesis.

The limitations of our study include that samples were mainly collected from hospitalized patients at the INER, located in Mexico City. This restricts the assessment of hRSV lineages circulation in other regions of Mexico and excludes the genetic characterization of viruses infecting individuals with mild disease. Also, the method used to sequence the complete viral genome excluded samples with low viral loads (Ct > 25) preventing the evaluation of potential associations between viral load and hRSV lineages. Finally, while substitutions previously related to resistance to monoclonal antibodies were identified, this study did not include functional assays to confirm their effect on the efficacy of preventive treatments such as Palivizumab or Nirsevimab.

## Supplementary Information

Below is the link to the electronic supplementary material.

Supplementary Material 1

Supplementary Material 2

Supplementary Material 3

## Data Availability

The genomic information generated during the current study is available in GISAID database. Sequences of hRSV-A and hRSV-B from Mexico were deposited in GISAID under accession number EPI_ISL_19500979- EPI_ISL_19501009 and EPI_ISL_19504707-EPI_ISL_19504788. GISAID Identifier: EPI_SET_250630bdDOI: https://doi.org/10.55876/gis8.250630bd Supplemental Table.

## References

[1] Li, Y. et al. Global, regional, and National disease burden estimates of acute lower respiratory infections due to respiratory syncytial virus in children younger than 5 years in 2019: a systematic analysis. *Lancet* **399** (10340), 2047–2064 (2022). 35598608 10.1016/S0140-6736(22)00478-0 PMC7613574

[2] Kenmoe, S. & Nair, H. The disease burden of respiratory syncytial virus in older adults. *Curr. Opin. Infect. Dis.* **37** (2), 129–136 (2024). 38197402 10.1097/QCO.0000000000001000 PMC10911257

[3] Melero, J. A., Mas, V. & McLellan, J. S. Structural, antigenic and Immunogenic features of respiratory syncytial virus glycoproteins relevant for vaccine development. *Vaccine* **35** (3), 461–468 (2017). 27692522 10.1016/j.vaccine.2016.09.045 PMC5189713

[4] Hamza, A. et al. Structural characterization of ectodomain G protein of respiratory syncytial virus and its interaction with Heparan sulfate: Multi-Spectroscopic and in Silico studies elucidating Host-Pathogen interactions. *Molecules* **26** (23), 7398 (2021). 34885979 10.3390/molecules26237398 PMC8658883

[5] Feldman, S. A., Hendry, R. M. & Beeler, J. A. Identification of a linear heparin binding domain for human respiratory syncytial virus attachment glycoprotein G. *J. Virol.* **73** (8), 6610–6617 (1999). 10400758 10.1128/jvi.73.8.6610-6617.1999 PMC112745

[6] McLellan, J. S., Ray, W. C. & Peeples, M. E. Structure and function of respiratory syncytial virus surface glycoproteins. *Curr. Top. Microbiol. Immunol.* **372**, 83–104 (2013). 24362685 10.1007/978-3-642-38919-1_4 PMC4211642

[7] Ruckwardt, T. J. The road to approved vaccines for respiratory syncytial virus. *NPJ Vaccines*. **8** (1), 138 (2023). 37749081 10.1038/s41541-023-00734-7 PMC10519952

[8] Yu, J. M., Fu, Y. H., Peng, X. L., Zheng, Y. P. & He, J. S. Genetic diversity and molecular evolution of human respiratory syncytial virus A and B. *Sci. Rep.* **11** (1), 12941 (2021). 34155268 10.1038/s41598-021-92435-1 PMC8217232

[9] Trento, A. et al. Major changes in the G protein of human respiratory syncytial virus isolates introduced by a duplication of 60 nucleotides. *J. Gen. Virol.* **84** (Pt 11), 3115–3120 (2003). 14573817 10.1099/vir.0.19357-0

[10] Eshaghi, A. et al. Genetic variability of human respiratory syncytial virus A strains Circulating in ontario: a novel genotype with a 72 nucleotide G gene duplication. *PLoS One*. **7** (3), e32807 (2012). 22470426 10.1371/journal.pone.0032807 PMC3314658

[11] Goya, S. et al. Standardized phylogenetic classification of human respiratory syncytial virus below the subgroup level. *Emerg. Infect. Dis.* **30** (8), 1631–1641 (2024). 39043393 10.3201/eid3008.240209 PMC11286072

[12] Wong-Chew, R. M. et al. Mexican interdisciplinary consensus on the diagnosis and preventive measures for respiratory syncytial virus infections. *Arch. Med. Res.* **56** (4), 103183 (2025). 39983633 10.1016/j.arcmed.2025.103183

[13] Vazquez-Pérez, J. A. et al. An amplicon-based protocol for whole-genome sequencing of human respiratory syncytial virus subgroup A. *Biol. Methods Protoc.* **9** (1), bpae007 (2024). 38371356 10.1093/biomethods/bpae007 PMC10873904

[14] Davina-Nunez, C., Perez-Castro, S., Godoy-Diz, M. & Regueiro-Garcia, B. Whole-Genome Amplification of Respiratory Syncytial Virus (RSV) using Illumina CovidSeq reagents for Next-G. https://www.protocols.io/view/whole-genome-amplification-of-respiratory-syncytia-cx3hxqj6 (2025).

[15] Briese, T. et al. Virome capture sequencing enables sensitive viral diagnosis and comprehensive Virome analysis. *mBio* **6** (5), e01491–e01415 (2015). 26396248 10.1128/mBio.01491-15 PMC4611031

[16] Mechikoff, M. A. et al. Application of Pan-Viral metagenomic sequencing on united States air force academy wastewater to uncover potential causes of acute gastroenteritis. *Mil Med.* **2024**, usae518 (2024). 10.1093/milmed/usae518 39545936

[17] Rambaut, A. FigTree [Internet] (2024, accessed 13 Mar 2024). http://tree.bio.ed.ac.uk/software/figtree/.

[18] Nextclade [Internet] (2015, accessed 15 Jan 2025). https://clades.nextstrain.org.

[19] Aksamentov, I., Roemer, C., Hodcroft, E. B. & Neher, R. A. Nextclade: clade assignment, mutation calling and quality control for viral genomes. *J. Open Source Softw.* **6** (67), 3773. 10.21105/joss.03773 (2021).

[20] Sutto-Ortiz, P., Eléouët, J. F., Ferron, F. & Decroly, E. Biochemistry of the respiratory syncytial virus L protein embedding RNA polymerase and capping activities. *Viruses* **15** (2), 341 (2023). 36851554 10.3390/v15020341 PMC9960070

[21] Rijsbergen, L. C. et al. Human respiratory syncytial virus subgroup A and B infections in nasal, bronchial, Small-Airway, and Organoid-Derived respiratory cultures. *mSphere* **6** (3), e00237–e00221 (2021). 33980679 10.1128/mSphere.00237-21 PMC8125053

[22] Hierholzer, J. C. et al. Subgrouping of respiratory syncytial virus strains from Australia and Papua new Guinea by biological and antigenic characteristics. *Arch. Virol.* **136** (1–2), 133–147 (1994). 8002781 10.1007/BF01538823

[23] Devincenzo, J. P. Natural infection of infants with respiratory syncytial virus subgroups A and B: a study of frequency, disease severity, and viral load. *Pediatr. Res.* **56** (6), 914–917 (2004). 15470202 10.1203/01.PDR.0000145255.86117.6A

[24] McGinley, J. P. et al. Clinical and viral factors associated with disease severity and subsequent wheezing in infants with respiratory syncytial virus infection. *J. Infect. Dis.* **226** (Suppl 1), S45–54 (2022). 35902389 10.1093/infdis/jiac163

[25] Tramuto, F. et al. Whole-Genome sequencing and genetic diversity of human respiratory syncytial virus in patients with Influenza-like illness in Sicily (Italy) from 2017 to 2023. *Viruses* **16** (6), 851 (2024). 38932144 10.3390/v16060851 PMC11209242

[26] Evans, D. et al. Genomic epidemiology of human respiratory syncytial virus, minnesota, USA, July 2023-February 2024. *Emerg. Infect. Dis.* **30** (11), 2414–2418 (2024). 39447178 10.3201/eid3011.241000 PMC11521169

[27] Wei, X. et al. Novel imported clades accelerated the RSV surge in beijing, china, 2023–2024. *J. Infect.* **89** (6), 106321 (2024). 39426631 10.1016/j.jinf.2024.106321

[28] Elena, S. F. & Sanjuán, R. Adaptive value of high mutation rates of RNA viruses: separating causes from consequences. *J. Virol.* **79** (18), 11555–11558 (2005). 16140732 10.1128/JVI.79.18.11555-11558.2005 PMC1212614

[29] Regoes, R. R., Hamblin, S. & Tanaka, M. M. Viral mutation rates: modelling the roles of within-host viral dynamics and the trade-off between replication fidelity and speed. *Proc. Biol. Sci.* **280** (1750), 20122047 (2013). 23135674 10.1098/rspb.2012.2047 PMC3574426

[30] Leemans, A. et al. Characterization of the role of N-glycosylation sites in the respiratory syncytial virus fusion protein in virus replication, syncytium formation and antigenicity. *Virus Res.* **266**, 58–68 (2019). 31004621 10.1016/j.virusres.2019.04.006

[31] Zimmer, G., Trotz, I. & Herrler, G. N-glycans of F protein differentially affect fusion activity of human respiratory syncytial virus. *J. Virol.* **75** (10), 4744–4751 (2001). 11312346 10.1128/JVI.75.10.4744-4751.2001 PMC114229

[32] Zhu, Q. et al. Natural polymorphisms and resistance-associated mutations in the fusion protein of respiratory syncytial virus (RSV): effects on RSV susceptibility to Palivizumab. *J. Infect. Dis.* **205** (4), 635–638 (2012). 22184728 10.1093/infdis/jir790

[33] Papenburg, J. et al. Molecular evolution of respiratory syncytial virus fusion gene, canada, 2006–2010. *Emerg. Infect. Dis.* **18** (1), 120–124 (2012). 22264682 10.3201/eid1801.110515 PMC3310097

[34] Wilkins, D. et al. Nirsevimab binding-site conservation in respiratory syncytial virus fusion glycoprotein worldwide between 1956 and 2021: an analysis of observational study sequencing data. *Lancet Infect. Dis.* **23** (7), 856–866 (2023). 36940703 10.1016/S1473-3099(23)00062-2

[35] Zhu, Q. et al. Prevalence and significance of substitutions in the fusion protein of respiratory syncytial virus resulting in neutralization escape from antibody MEDI8897. *J. Infect. Dis.* **218** (4), 572–580 (2018). 29617879 10.1093/infdis/jiy189

[36] Zhu, Q. et al. A highly potent extended half-life antibody as a potential RSV vaccine surrogate for all infants. *Sci. Transl Med.* **9** (388), eaaj1928 (2017). 28469033 10.1126/scitranslmed.aaj1928

[37] Nuttens, C. et al. Differences between RSV A and RSV B subgroups and implications for pharmaceutical preventive measures. *Infect. Dis. Ther.* **13** (8), 1725–1742 (2024). 38971918 10.1007/s40121-024-01012-2 PMC11266343

[38] Hause, A. M. et al. Sequence variability of the respiratory syncytial virus (RSV) fusion gene among contemporary and historical genotypes of RSV/A and RSV/B. *PLoS One*. **12** (4), e0175792 (2017). 28414749 10.1371/journal.pone.0175792 PMC5393888

[39] Johnson, S. M. et al. Respiratory syncytial virus uses CX3CR1 as a receptor on primary human airway epithelial cultures. *PLoS Pathog*. **11** (12), e1005318 (2015). 26658574 10.1371/journal.ppat.1005318 PMC4676609

[40] Cortjens, B. et al. Broadly reactive Anti-Respiratory syncytial virus G antibodies from exposed individuals effectively inhibit infection of primary airway epithelial cells. *J. Virol.* **91** (10), e02357–e02316 (2017). 28275185 10.1128/JVI.02357-16 PMC5411575

[41] Bergeron, H. C. et al. Immunogenicity and protective efficacy of an RSV G S177Q central conserved domain nanoparticle vaccine. *Front. Immunol.* **14**, 1215323 (2023). 37457705 10.3389/fimmu.2023.1215323 PMC10338877

[42] Bergeron, H. C., Murray, J., Nuñez Castrejon, A. M., DuBois, R. M. & Tripp, R. A. Respiratory syncytial virus (RSV) G protein vaccines with central conserved domain mutations induce CX3C-CX3CR1 blocking antibodies. *Viruses* **13** (2), 352 (2021). 33672319 10.3390/v13020352 PMC7926521

[43] Li, W. et al. Disease severity of respiratory syncytial virus (RSV) infection correlate to a novel set of five amino acid substitutions in the RSV attachment glycoprotein (G) in China. *Virus Res.* **281**, 197937 (2020). 32194139 10.1016/j.virusres.2020.197937

[44] Morin, B., Kranzusch, P. J., Rahmeh, A. A. & Whelan, S. P. J. The polymerase of negative-stranded RNA viruses. *Curr. Opin. Virol.* **3** (2), 103–110 (2013). 23602472 10.1016/j.coviro.2013.03.008 PMC4159711

[45] Tiong-Yip, C. L. et al. Characterization of a respiratory syncytial virus L protein inhibitor. *Antimicrob. Agents Chemother.* **58** (7), 3867–3873 (2014). 24777090 10.1128/AAC.02540-14 PMC4068518

[46] Yu, X. et al. Structural and mechanistic insights into the Inhibition of respiratory syncytial virus polymerase by a non-nucleoside inhibitor. *Commun. Biol.* **6** (1), 1074 (2023). 37865687 10.1038/s42003-023-05451-4 PMC10590419

[47] Atchison, E. B. et al. Interaction between the matrix protein and the polymerase complex of respiratory syncytial virus. *Viruses* **16** (12), 1881 (2024). 39772190 10.3390/v16121881 PMC11680393

