# Supplementary material for: Molecular characterization of human respiratory syncytial virus in Mexico (season 2023–2024) through whole-genome sequencing
Source: Sci Rep. 2025 Jul 28;15:27382. doi: 10.1038/s41598-025-13061-9 (PMC12304177; doi:10.1038/s41598-025-13061-9)

Table S1. Coverage and coverage depth data of hRSV-A and hRSV-B Mexico 2023-2024

| **Name** | **GISAID ID** | **Coverage** | **Coverage depth (median)** | **Ambiguous bases (Ns)** |
| --- | --- | --- | --- | --- |
| hRSV/A/Mexico/CMX_INER-049/2023 | EPI_ISL_19504724 | 98.3 | 80 | 4 |
| hRSV/A/Mexico/CMX_INER-050/2023 | EPI_ISL_19504725 | 100 | 200 | 0 |
| hRSV/A/Mexico/CMX_INER-051/2023 | EPI_ISL_19504726 | 100 | 811 | 0 |
| hRSV/A/Mexico/CMX_INER-052/2023 | EPI_ISL_19504727 | 100 | 212 | 0 |
| hRSV/A/Mexico/CMX_INER-053/2023 | EPI_ISL_19504728 | 100 | 638 | 0 |
| hRSV/A/Mexico/CMX_INER-054/2023 | EPI_ISL_19504729 | 98.5 | 691 | 0 |
| hRSV/A/Mexico/CMX_INER-055/2023 | EPI_ISL_19504730 | 97.3 | 20 | 126 |
| hRSV/A/Mexico/CMX_INER-056/2023 | EPI_ISL_19504731 | 98.7 | 279 | 24 |
| hRSV/A/Mexico/CMX_INER-057/2023 | EPI_ISL_19504732 | 99.3 | 334 | 102 |
| hRSV/A/Mexico/CMX_INER-058/2023 | EPI_ISL_19504733 | 98.1 | 66 | 29 |
| hRSV/A/Mexico/CMX_INER-059/2023 | EPI_ISL_19504734 | 100 | 893 | 0 |
| hRSV/A/Mexico/CMX_INER-060/2023 | EPI_ISL_19504735 | 100 | 739 | 2 |
| hRSV/A/Mexico/CMX_INER-061/2024 | EPI_ISL_19504736 | 95.6 | 25 | 395 |
| hRSV/A/Mexico/CMX_INER-062/2023 | EPI_ISL_19504737 | 99.8 | 28 | 24 |
| hRSV/A/Mexico/CMX_INER-063/2023 | EPI_ISL_19504738 | 98.3 | 72 | 9 |
| hRSV/A/Mexico/CMX_INER-064/2023 | EPI_ISL_19504739 | 97.9 | 22 | 64 |
| hRSV/A/Mexico/CMX_INER-065/2023 | EPI_ISL_19504741 | 96.2 | 31 | 318 |
| hRSV/A/Mexico/CMX_INER-066/2023 | EPI_ISL_19504742 | 98.9 | 547 | 1 |
| hRSV/A/Mexico/CMX_INER-067/2023 | EPI_ISL_19504743 | 98 | 185 | 51 |
| hRSV/A/Mexico/CMX_INER-068/2023 | EPI_ISL_19504744 | 98.5 | 1284 | 0 |
| hRSV/A/Mexico/CMX_INER-069/2023 | EPI_ISL_19504745 | 98.4 | 208 | 3 |
| hRSV/A/Mexico/CMX_INER-070/2023 | EPI_ISL_19504746 | 98.5 | 1257 | 0 |
| hRSV/A/Mexico/CMX_INER-071/2023 | EPI_ISL_19504747 | 98.9 | 773 | 0 |
| hRSV/A/Mexico/CMX_INER-072/2023 | EPI_ISL_19504748 | 98.6 | 1634 | 0 |
| hRSV/A/Mexico/CMX_INER-073/2023 | EPI_ISL_19504749 | 99 | 704 | 1 |
| hRSV/A/Mexico/CMX_INER-074/2023 | EPI_ISL_19504750 | 98.8 | 565 | 50 |
| hRSV/A/Mexico/CMX_INER-075/2023 | EPI_ISL_19504751 | 99.2 | 890 | 0 |
| hRSV/A/Mexico/CMX_INER-076/2023 | EPI_ISL_19504752 | 98.5 | 581 | 0 |
| hRSV/A/Mexico/CMX_INER-077/2023 | EPI_ISL_19504753 | 98.1 | 409 | 35 |
| hRSV/A/Mexico/CMX_INER-078/2023 | EPI_ISL_19504754 | 98.4 | 247 | 3 |
| hRSV/A/Mexico/CMX_INER-079/2023 | EPI_ISL_19504755 | 98.3 | 168 | 13 |
| hRSV/A/Mexico/CMX_INER-080/2023 | EPI_ISL_19504756 | 98.4 | 398 | 0 |
| hRSV/A/Mexico/CMX_INER-081/2023 | EPI_ISL_19504757 | 97.8 | 50 | 77 |
| hRSV/A/Mexico/CMX_INER-082/2023 | EPI_ISL_19504758 | 99.3 | 731 | 18 |
| hRSV/A/Mexico/CMX_INER-083/2023 | EPI_ISL_19504759 | 97.8 | 48 | 75 |
| hRSV/A/Mexico/CMX_INER-084/2024 | EPI_ISL_19504760 | 98 | 374 | 54 |
| hRSV/A/Mexico/CMX_INER-085/2023 | EPI_ISL_19504761 | 99.1 | 870 | 0 |
| hRSV/A/Mexico/CMX_INER-086/2023 | EPI_ISL_19504762 | 98.3 | 456 | 21 |
| hRSV/A/Mexico/CMX_INER-087/2023 | EPI_ISL_19504763 | 98.4 | 552 | 19 |
| hRSV/A/Mexico/CMX_INER-088/2023 | EPI_ISL_19504764 | 97.4 | 28 | 0 |
| hRSV/A/Mexico/CMX_INER-089/2023 | EPI_ISL_19504765 | 92.9 | 72 | 0 |
| hRSV/A/Mexico/CMX_INER-090/2023 | EPI_ISL_19504766 | 100 | 1746 | 0 |
| hRSV/A/Mexico/CMX_INER-091/2023 | EPI_ISL_19504767 | 99.3 | 933 | 100 |
| hRSV/A/Mexico/CMX_INER-092/2023 | EPI_ISL_19504768 | 100 | 1820 | 0 |
| hRSV/A/Mexico/CMX_INER-093/2023 | EPI_ISL_19504769 | 97.2 | 107 | 0 |
| hRSV/A/Mexico/CMX_INER-094/2023 | EPI_ISL_19504770 | 87.6 | 20 | 0 |
| hRSV/A/Mexico/CMX_INER-095/2023 | EPI_ISL_19504771 | 100 | 1386 | 0 |
| hRSV/A/Mexico/CMX_INER-096/2023 | EPI_ISL_19504772 | 100 | 1303 | 0 |
| hRSV/A/Mexico/CMX_INER-097/2023 | EPI_ISL_19504773 | 100 | 2068 | 0 |
| hRSV/A/Mexico/CMX_INER-098/2023 | EPI_ISL_19504774 | 98 | 77 | 0 |
| hRSV/A/Mexico/CMX_INER-099/2023 | EPI_ISL_19504775 | 100 | 266 | 0 |
| hRSV/A/Mexico/CMX_INER-100/2023 | EPI_ISL_19504776 | 100 | 1752 | 0 |
| hRSV/A/Mexico/CMX_INER-101/2023 | EPI_ISL_19504777 | 99 | 472 | 0 |
| hRSV/A/Mexico/CMX_INER-102/2023 | EPI_ISL_19504778 | 100 | 2677 | 0 |
| hRSV/A/Mexico/CMX_INER-103/2023 | EPI_ISL_19504779 | 99.3 | 1080 | 0 |
| hRSV/A/Mexico/CMX_INER-104/2023 | EPI_ISL_19504780 | 99.7 | 786 | 0 |
| hRSV/A/Mexico/CMX_INER-105/2023 | EPI_ISL_19504781 | 100 | 1707 | 0 |
| hRSV/A/Mexico/CMX_INER-106/2023 | EPI_ISL_19504782 | 100 | 981 | 0 |
| hRSV/A/Mexico/CMX_INER-107/2023 | EPI_ISL_19504783 | 89.7 | 70 | 0 |
| hRSV/A/Mexico/CMX_INER-108/2023 | EPI_ISL_19504784 | 100 | 2002 | 0 |
| hRSV/A/Mexico/CMX_INER-109/2023 | EPI_ISL_19504785 | 81.9 | 20 | 0 |
| hRSV/A/Mexico/CMX_INER-110/2023 | EPI_ISL_19504786 | 97.1 | 1543 | 0 |
| hRSV/A/Mexico/CMX_INER-111/2023 | EPI_ISL_19504787 | 100 | 1883 | 0 |
| hRSV/A/Mexico/CMX_INER-112/2023 | EPI_ISL_19504788 | 90.7 | 564 | 0 |
| hRSV/B/Mexico/CMX_INER-03/2023 | EPI_ISL_19500981 | 100 | 1808 | 0 |
| hRSV/B/Mexico/CMX_INER-04/2023 | EPI_ISL_19500982 | 100 | 1086 | 0 |
| hRSV/B/Mexico/CMX_INER-05/2023 | EPI_ISL_19500983 | 99.2 | 114 | 120 |
| hRSV/B/Mexico/CMX_INER-06/2023 | EPI_ISL_19500984 | 99.9 | 228 | 13 |
| hRSV/B/Mexico/CMX_INER-07/2023 | EPI_ISL_19500985 | 98.9 | 90 | 0 |
| hRSV/B/Mexico/CMX_INER-08/2023 | EPI_ISL_19500986 | 99.8 | 90 | 27 |
| hRSV/B/Mexico/CMX_INER-09/2023 | EPI_ISL_19500987 | 100 | 1750 | 1 |
| hRSV/B/Mexico/CMX_INER-10/2023 | EPI_ISL_19500988 | 100 | 829 | 0 |
| hRSV/B/Mexico/CMX_INER-11/2023 | EPI_ISL_19500989 | 99.8 | 90 | 37 |
| hRSV/B/Mexico/CMX_INER-12/2023 | EPI_ISL_19500990 | 99.8 | 90 | 27 |
| hRSV/B/Mexico/CMX_INER-13/2023 | EPI_ISL_19500991 | 99.4 | 90 | 90 |
| hRSV/B/Mexico/CMX_INER-14/2023 | EPI_ISL_19500992 | 99.8 | 95 | 15 |
| hRSV/B/Mexico/CMX_INER-15/2023 | EPI_ISL_19500993 | 100 | 461 | 1 |
| hRSV/B/Mexico/CMX_INER-16/2023 | EPI_ISL_19500994 | 100 | 698 | 1 |
| hRSV/B/Mexico/CMX_INER-17/2023 | EPI_ISL_19500995 | 99.8 | 99 | 31 |
| hRSV/B/Mexico/CMX_INER-18/2023 | EPI_ISL_19500996 | 99.6 | 86 | 13 |
| hRSV/B/Mexico/CMX_INER-19/2023 | EPI_ISL_19500997 | 99.9 | 85 | 7 |
| hRSV/B/Mexico/CMX_INER-20/2023 | EPI_ISL_19500998 | 100 | 113 | 2 |
| hRSV/B/Mexico/CMX_INER-21/2023 | EPI_ISL_19500999 | 99.9 | 156 | 9 |
| hRSV/B/Mexico/CMX_INER-22/2023 | EPI_ISL_19501000 | 99.4 | 25 | 15 |
| hRSV/B/Mexico/CMX_INER-23/2023 | EPI_ISL_19501001 | 99.9 | 110 | 16 |
| hRSV/B/Mexico/CMX_INER-24/2023 | EPI_ISL_19501002 | 100 | 2025 | 0 |
| hRSV/B/Mexico/CMX_INER-25/2023 | EPI_ISL_19501003 | 100 | 738 | 0 |
| hRSV/B/Mexico/CMX_INER-26/2023 | EPI_ISL_19501004 | 100 | 1146 | 0 |
| hRSV/B/Mexico/CMX_INER-27/2023 | EPI_ISL_19501005 | 100 | 264 | 3 |
| hRSV/B/Mexico/CMX_INER-28/2024 | EPI_ISL_19501006 | 100 | 125 | 3 |
| hRSV/B/Mexico/CMX_INER-29/2024 | EPI_ISL_19501007 | 97.9 | 132 | 11 |
| hRSV/B/Mexico/CMX_INER-30/2024 | EPI_ISL_19501008 | 99.9 | 112 | 18 |
| hRSV/B/Mexico/CMX_INER-31/2024 | EPI_ISL_19501009 | 99.7 | 444 | 0 |

Median=398, IQR=801.5

Figure S1. High coverage depth hRSV-B, Sample GISAID ID:EPI_ISL_19501002


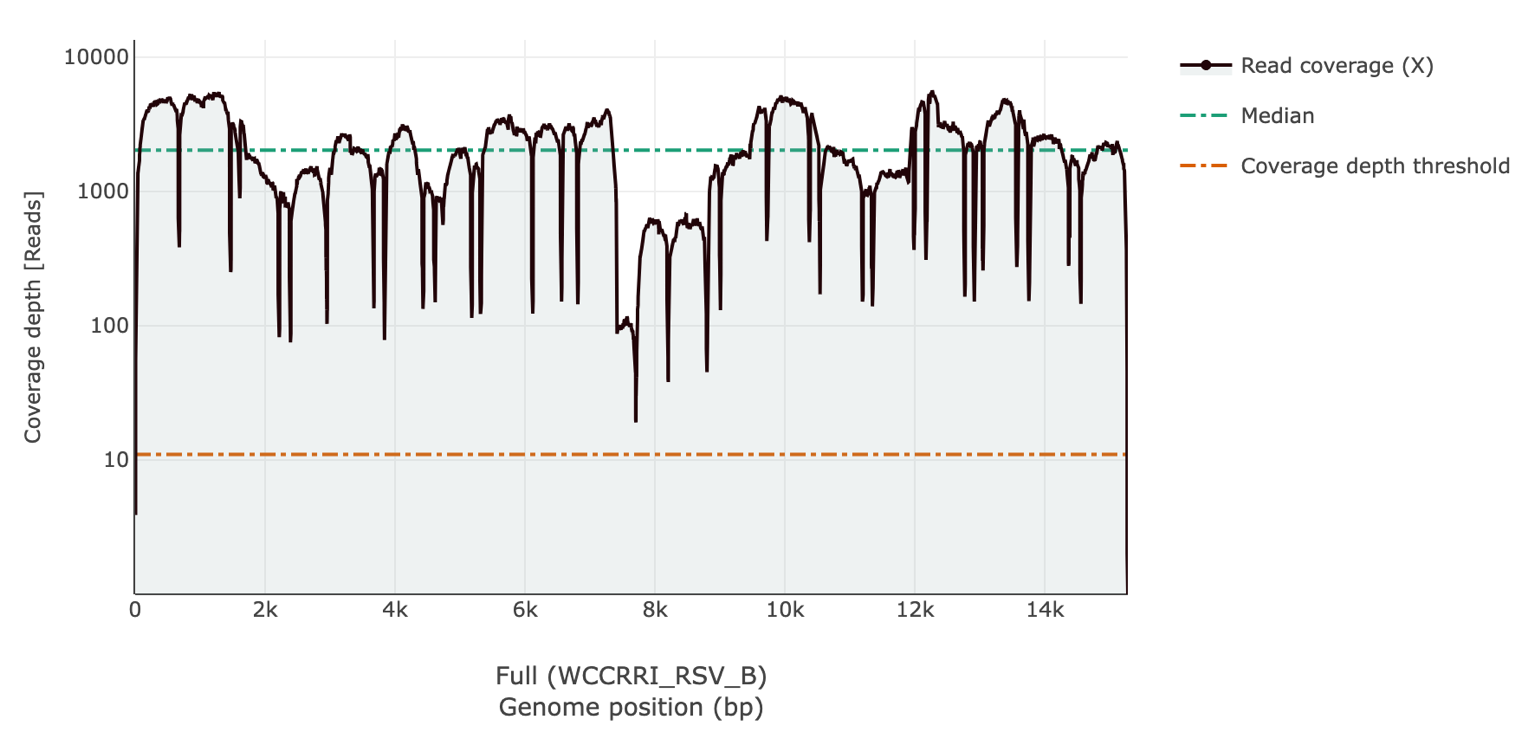


Figure S2. Low coverage depth hRSV-B, Sample, GISAID ID: EPI_ISL_19501007


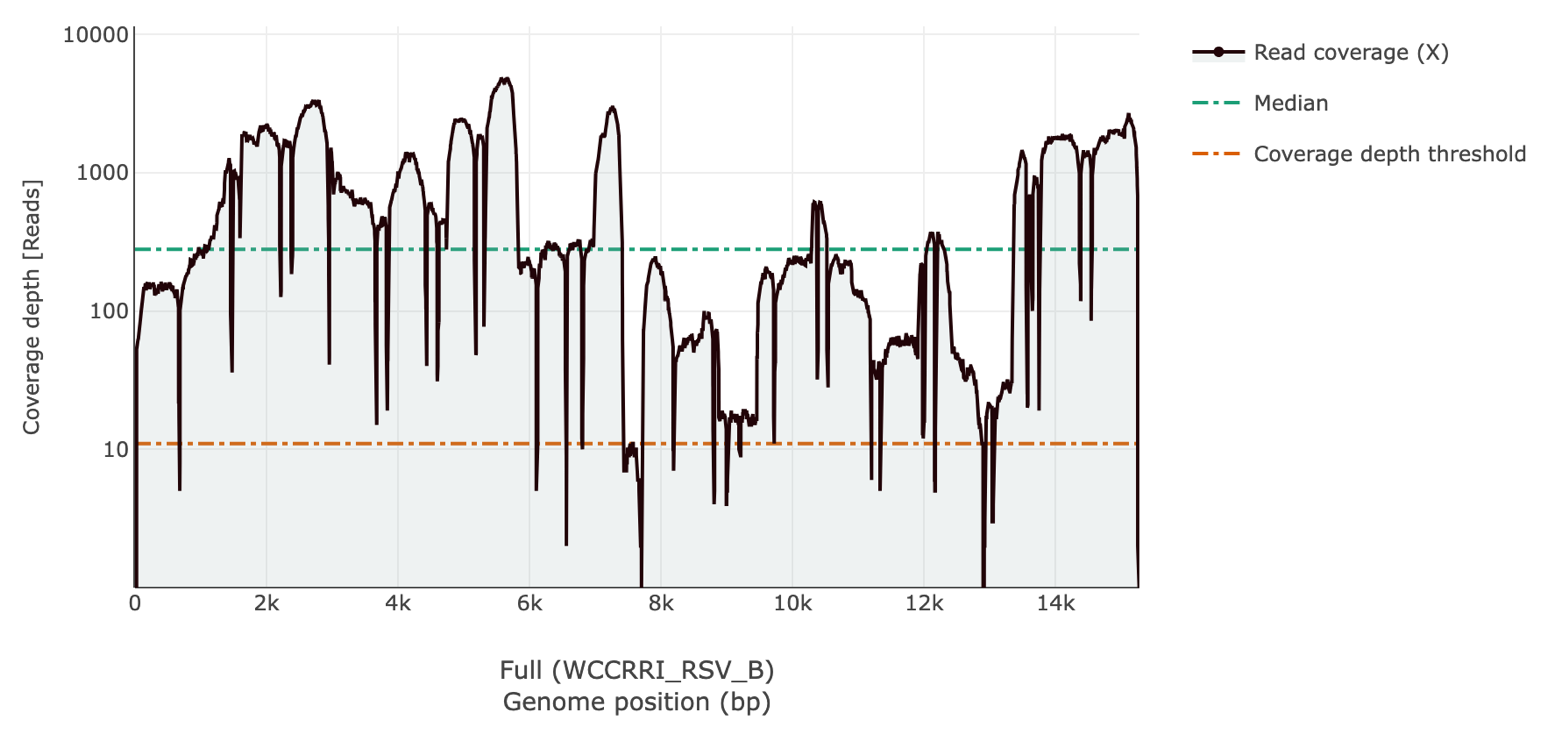


Figure S3. High coverage depth hRSV-A, Sample, GISAID ID: EPI_ISL_19504781


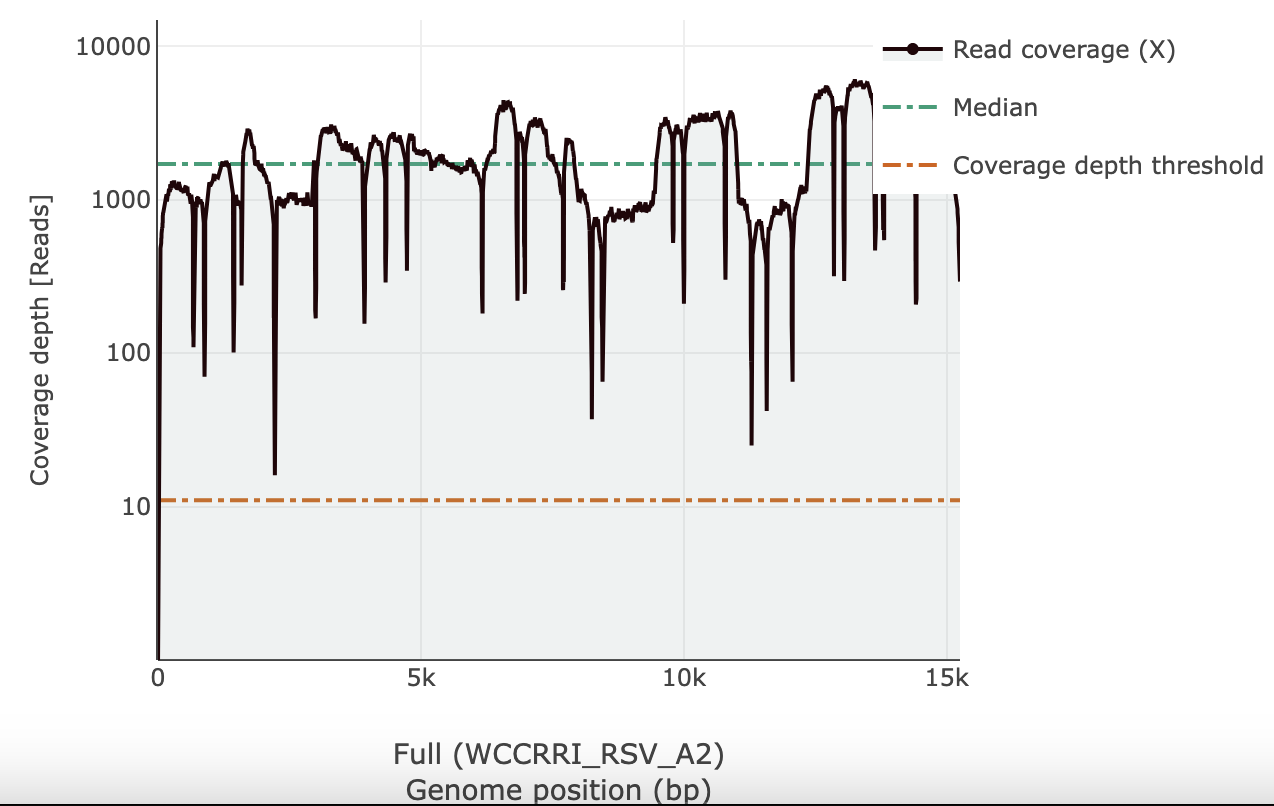


Figure S4. Low coverage depth hRSV-A, Sample, GISAID ID: EPI_ISL_19504765


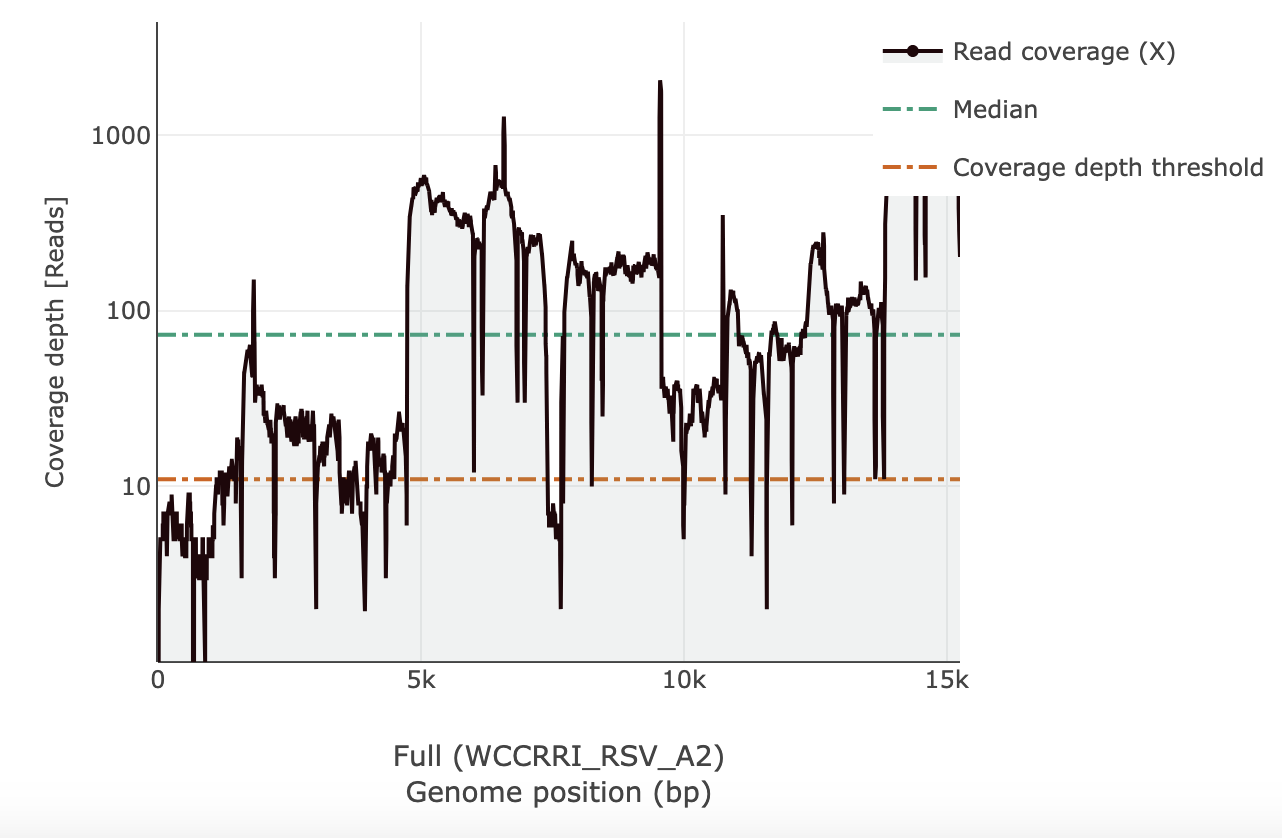

Supplement: Supplementary file 1 — Supplementary Material 1 [file 41598_2025_13061_MOESM1_ESM.docx]
