# Supplementary material for: Molecular characterization of human respiratory syncytial virus in Mexico (season 2023–2024) through whole-genome sequencing
Source: Sci Rep. 2025 Jul 28;15:27382. doi: 10.1038/s41598-025-13061-9 (PMC12304177; doi:10.1038/s41598-025-13061-9)
Supplement: Supplementary file 3 — Supplementary Material 3 [file 41598_2025_13061_MOESM3_ESM.pdf]

SUPPLEMENTAL TABLE

Data Availability

GISAID Identifier:	EPI_SET_250630bd
DOI:	https://doi.org/10.55876/gis8.250630bd

All genome sequences and associated metadata in this dataset are published in GISAID's EpiRSV database. To view the contributors of each individual sequence with details such as accession number, Virus name, Collection date, Originating Lab and Submitting Lab and the list of Authors, visit EPI_SET_250630bd

Data Snapshot

EPI_SET_250630bd is composed of 112 individual genome sequences.
The collection dates range from 2022-09-28 to 2024-02-11;
Data were collected in 1 countries and territories.
